# Effects of cyclin-dependent kinase 8 specific siRNA on the proliferation and apoptosis of colon cancer cells

**DOI:** 10.1186/1756-9966-30-109

**Published:** 2011-11-22

**Authors:** Song-Bing He, Yin Yuan, Lei Wang, Min-Jing Yu, Yi-Bei Zhu, Xing-Guo Zhu

**Affiliations:** 1 Department of General Surgery, the First Affiliated Hospital of Soochow University, 215006 Suzhou, Jiangsu Province, China; 2 Department of immunology, Soochow University, 215006 Suzhou, Jiangsu Province, China

## Abstract

**Background:**

To investigate the expression of cyclin-dependent kinase 8 (CDK8) and β-catenin in colon cancer and evaluate the role of CDK8 in the proliferation, apoptosis and cell cycle progression of colon cancer cells, especially in HCT116 cell line.

**Methods:**

Colon cancer cell line HCT116 was transfected with small interfering RNA (siRNA) targeting on CDK8. After CDK8-siRNA transfection, mRNA and protein expression levels of CDK8 and β-catenin were determined by reverse transcriptase-polymerase chain reaction (RT-PCR) and Western blot assay in HCT116 cells. Cell proliferation was measured by 3-(4, 5-Dimethylthiazol-2-yl)-2, 5-diphenyltetrazolium bromide Methylthiazolyl tetrazolium (MTT) assay, and cell cycle distribution and apoptosis were analyzed by flow cytometry analysis (FACS). CDK8 and β-catenin protein levels were also examined by real-time PCR and immunohistochemistry (IHC) in colon cancer tissues and adjacent normal tissues.

**Results:**

After CDK8 specific siRNA transfection, mRNA and protein expression levels of CDK8 and β-catenin in HCT116 cells were noticeably decreased (*P *< 0.05). CDK8 specific siRNA transfection inhibited HCT116 cells' proliferation and facilitated their apoptosis significantly (*P *< 0.05). In addition, the proportion of HCT116 cells in the G0/G1 phase was remarkably increased after CDK8-siRNA transfection (*P *< 0.05). The expression levels of CDK8 and β-catenin in adjacent normal tissues were lower than in tumor tissues (*P *< 0.05). Moreover, the expression of CDK8 was correlated with the expression of β-catenin in both tumor and adjacent normal tissues (*P *< 0.05).

**Conclusions:**

CDK8 and β-catenin were expressed in colon cancer at a high frequency. CDK8 specific siRNA transfection down-regulated the expression of CDK8 in colon cancer cells, which was also associated with a decrease in the expression of β-catenin Moreover, CDK8 specific siRNA inhibited the proliferation of colon cancer cells, promoted their apoptosis and arrested these cells in the G0/G1 phase. Interference of CDK8 might be an effective strategy through β-catenin regulation of colon cancer.

## Background

Colon cancer is a result of an evolving process characterized by alterations of multiple genes and dysregulated cell signal transduction pathways. It has been well known that mutations of key genes in the Wnt/β-catenin signaling pathway play an important role in the occurrence and development of colon cancer [1,2]. Under physiological conditions, Wnt contributes to the stabilization of β-catenin. Once stabilized, β-catenin accumulates and migrates to the nucleus. On the other hand, when the level of β-catenin within the cytoplasm surpasses the threshold, the molecule migrates to the nucleus, binds to the transcription factor, and exposes the promoters of downstream genes, such as c-myc, cyclinD1, survivin, gastrin, vascular endothelial growth factor (VEGF), and associated exchange factors (Asef). Finally, these activated genes contribute to abnormal cellular proliferation [3,4].

Cyclin-dependent kinase (CDK) 8 is located in chromosome 13q12.13 and is a member of the CDK family [5,6]. CDK is classified as a serine-threonine protein kinase, and ten of its members have been identified in the CDK family so far, where these members have some homology to a certain extent. CDK has a catalytic subunit that is activated in the presence of a regulatory subunit provided by cyclin [7], which leads to the formation of a mediator complex together with MDE12 and MED13. The mediator complex can bind to RNA polymerase II, which participates in eukaryotic gene transcription such as the transcription of the β-catenin signaling pathway. Taken together, CDK8 plays an important regulatory role in cell cycle control and cell growth at the transcription level and it is proposed to be a proto-oncogene in human colon cancer [8-10]. As far as we know, studies on the role of CDK8 in the proliferation, apoptosis and cell cycle progression of colon cancer cells are still insufficient [11]. RNA interference (RNAi) has emerged as a powerful tool to induce lose-of-function phenotypes by post-transcriptional silencing of gene expression [12,13]. In the present study, CDK8 specific interference was designed and transfected into a colon cancer cell line HCT116. The effect of small interfering RNA (siRNA) silencing of CDK8 on the growth of colon cancer cells was investigated. In addition, we verified the mRNA and protein expression levels of CDK8 and β-catenin in colon cancer tissues.

## Methods

### Major reagents

Rabbit anti-human CDK8 antibody, rabbit anti-human β-catenin antibody, and rat anti-human β-actin antibody were purchased from Chemicon (USA). Lipofectin2000 was provided by Invitrogen (USA). RT-PCR kits were purchased from Fermentas (USA). Annexin V apoptosis kit (Keygentec, China) and siRNA-CDK8 (Genepharma, China) were used in the present study.

### Cell culture

The human colon cancer cell line, HCT116 cell line was purchased from Shanghai Cell Biology Institutes, Chinese Academy of Sciences (Shanghai, China). HCT116 cell line was seeded in 6-well plate at a density of 1.5 × 10^5^/well and maintained in RPMI1640 (Invitrogen, USA) supplemented with 10% fetal bovine serum (FBS). All cells were cultured at 37°C in a humidified atmosphere containing 5% CO_2_.

### Transfection with CDK8-siRNA

CDK8 siRNA sequence 5'-AUAUAAUAGUGACUUCACCAUUCCCTT-3' (S) 5'-GGGAAUGGUGAAGUVAVUAUUAUAUTT-3' (AS) and scrambled siRNA sequence 5'-UUCUCCGAACGUGUCACGUTT-3' (S) 5'-ACGUGACACGUUCGGAGAATT-3' (AS) were designed and synthesized by Genepharma (Shanghai, China). HCT116 cells (1.5 × 10^5^) were divided into three groups: (a) siRNA-CDK8 group, (b) scrambled siRNA group, and (c) non-siRNA control group. One hour before transfection, the medium was replaced with 1.5 ml of serum free Opti-MEM. Then, the following reagents were mixed as follows: (a) 4 μl of Lipofectin2000 were added to 250 μl of Opti-MEM, and the mixture was kept at room temperature for 5 min; (b) 4 μl of siRNA stock solution were added to 250 μl of Opti-MEM (siRNA dilution); and (c) the above two mixtures were then mixed and kept at room temperature for 20 min (siRNA-Lipofectin2000 mixture). After shaking, this siRNA-Lipofectin2000 mixture was then added to a 6-well plate (1.5 ml of Opti-MEM in each well). Six hours later, the medium was replaced with complete medium. Our previous study confirmed that we obtained the maximal transfection efficacy when the ratio of Lipofectin2000 to siRNA was 4 μl:4 μl.

### MTT assay

Six hours after transfection, HCT116 cells were digested, re-suspended and seeded in a 96-well culture plate. After 24, 48 and 72 h of incubation, cells were stained with 20 μl 3-(4, 5-Dimethylthiazol-2-yl)-2, 5-diphenyltetrazolium bromide Methylthiazolyl tetrazolium (MTT) solution (5 mg/ml) at 37°C for 4 h and subsequently made soluble in 150 μl of DMSO. Absorbance (A) was measured at 490 nm with an automated plate reader. Each sample was triplicated and the experiment was repeated three times. Cell growth curves were calculated as mean values of each group.

### Flow cytometric analysis

Cells were trypsinized and centrifuged at 1500 rpm/min for 5 min at 48 h after transfection. Cells were harvested and washed with Phosphate Buffered Saline (PBS) twice. Reagents for apoptosis detection were added, and then cells were incubated in dark for 30 min and subjected to flow cytometry analysis (FACS). Additionally, cells were collected, washed with PBS, fixed with 75% ethanol at-20°C overnight, and centrifuged at 1500 rpm/min for 5 min. Then, ethanol was removed and cells were washed with PBS twice. Propidium iodide (PI) and 500 μl of RNAse were added, and then cells were incubated in dark at 4°C for 60 min. Lastly, cells were subjected to cell cycle analysis by FACS.

### Gene expression analysis (RT-PCR and real-time PCR)

The mRNA expression of CDK8 and β-catenin in HCT116 cells after CDK8-siRNA transfection were quantified by RT-PCR. Total RNA was extracted from cells with Trizol and subjected to reverse transcription into cDNA. CDK8 and β-catenin were amplified from the cDNA by RT-PCR. The PCR conditions consisted of 5 min at 94°C one cycle, 30 s at 94°C, 40 s at 55°C, 45 s at 72°C, and 7 min at 72°C 40 cycles. The primer sequences were as follows: 5'-TCACCTTTGAAGCCTTTAGC-3' (forward) and 5'-CTGATGTAGGAAGTGGGTCT-3' (reverse) for CDK8; 5'-TGCCAAGTGGGTGGTATAGAG-3' (forward) and 5'-TGGGATGGTGGGTGTAAGAG-3' (reverse) for β-catenin; 5'CTGGGACGACATGGAGAAAA3' (forward) and 5'AAGGAAGGCTGGAAGAGTGC3' (reverse) for β-actin.

The mRNA expression of CDK8 and β-catenin in colon cancer samples (n = 12) were quantified by real-time PCR. Informed consent was obtained from all the patients, and research protocols were approved by Independent Ethics Committee (IEC) of our hospital. A total of 200 mg of tumor or adjacent normal tissues (at least 2 cm distant from the tumor site) were homogenized in liquid nitrogen. Total RNA was extracted and reverse transcribed into cDNA, which was then used for amplification of CDK8 and β-catenin. The real time PCR conditions consisted of 1 cycle at 94°C for 10 min followed by 40 cycles at 94°C for 30 s, at 55°C for 30 s, and at 72°C for 30 s. GAPDH was employed as an internal standard. The primer sequences were as follows: 5'-GAGCGGGTCGAGGACCTGTTTGAAT-3' (forward) and 5'-ACATGCCGACATAGAGATCCCAGTTCCTTC-3' (reverse) for CDK8; 5'-TGCCAAGTGGGTGGTATAGAG-3' (forward) and 5'-TGGGATGGTGGGTGTAAGAG-3' (reverse) for β-catenin; 5'AGGGGCCATCCACAGTCTTC3' (forward) and 5' AGAAGGCTGGGGCTCATTTG 3 (reverse) for GAPDH. The 2 ^-ΔΔCT ^method was applied to analyze the relative changes in gene expression.

### Western blot analysis

As described previously [14], following 72 h of transfection, total protein was extracted from HCT116 cells and subjected to SDS-PAGE. Protein concentrations were transferred onto PVDF membrane, then membranes were blocked and incubated with rabbit anti-human CDK8 (1:1000) or β-catenin antibody (1:1000) at 4°C overnight. After 3 washes with TBS-T solution for 10 min, the membranes underwent hybridization with a goat anti-rabbit IgG secondary antibody (1:1000) at 37°C for 1 h. After further washing, CDK8 and β-catenin levels were visualized using an ECL chemiluminescence kit.

### Immunohistochemistry

The protein expression of CDK8 and β-catenin in 47 tumor tissues and adjacent normal tissues were detected by IHC. Samples were fixed in 10% neutral formaldehyde, embedded in paraffin, and sliced. Briefly, the paraffin-embedded tissues were serially cut into 4 μm sections, dewaxed, and rehydrated. Sections were then blocked with peroxide and non-immune animal serum and incubated sequentially with rat anti-human CDK8 and β-catenin (1:1000), and biotin-labeled goat anti-rabbit IgG (1:1000). Finally, the sections were stained with DBA, counterstained with hematoxylin, dehydrated, cleared in xylene, and fixed.

Histological assessment was performed as described previously [15]. Immunostaining was independently examined by two clinical pathologists who were unaware of the patient outcome. Five high-power fields (400 × magnification) were randomly counted for each section. The brown staining on the cytoplasm was read as positive reactivity for CDK8 and β-catenin. The presence of brown colored granules on the cytoplasm was taken as a positive signal, and was divided by color intensity into not colored, light yellow, brown, tan and is recorded as 0, 1, 2, 3, respectively. We also choose five high-power fields from each slice and score them. Positive cell rate of < 25% was a score of 1, positive cell rate of 25~50% was a score of 2, positive cell rate of 51~75% was a score of 3, positive cell rate of > 75% was a score of 4. The final score was determined by multiplying positive cell rate and score values: 0 was equal to negative ( - ), 1~4 was equal to weakly positive (+), 5~8 was equal to moderate positive(+ +), 9~12 was equal to strongly positive (+ + +).

### Statistical analysis

All data were presented as means ± standard deviation (SD). A Student's t-test was used for comparisons between groups, and F test was applied for correlation analyses. Statistical analysis was performed with SPSS 13.0 statistic software package. *P *values < 0.05 were considered to be statistically significant.

## Results

### Effect of CDK8-siRNA transfection on CDK8 and β-catenin expression in HCT116 cells

**Figure 1:**
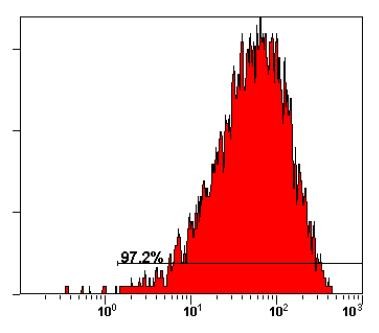
**Transfection efficiency determined by flow cytometry**. The transfection efficiency was 97.2% 6 h after transfecting with CDK8-siRNA of HCT116. The ratio of Lipofectin 2000 to siRNA was 4 μL: 4 μL, and the concentration of CDK8-siRNA is 80 pmol/L.

Six hours after CDK8-siRNA transfection, the transfection efficiency was detected by FACS. Our previous study confirmed that the maximal transfection efficacy could be obtained when the ratio of Lipofectin 2000 to siRNA was 4 μL: 4 μL. (Figure 1)

**Figure 2:**
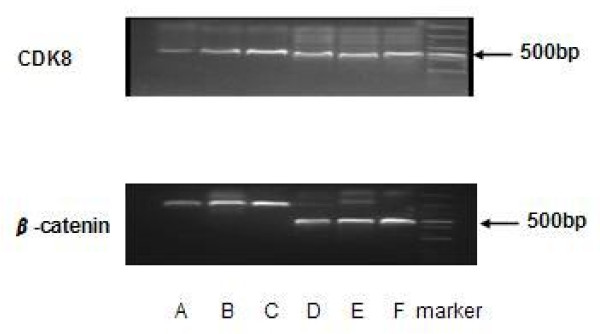
**CDK8 and β-catenin mRNA expression of CDK-siRNA transfected HCT116 cells detected by RT-PCR**. 48 h later of CDK8-siRNA transfection, RT-PCR was performed to detect CDK8 and β-catenin mRNA expression. A: CDK8-siRNA group; B: scrambled siRNA group; C: non-siRNA group; D, E and F represented corresponding internal reference, and M: marker. Results are given as average value of the gray in three target genes and interal controls from three independent experiments.

Forty-eight hours later of CDK8-siRNA transfection, RT-PCR was performed to detect CDK8 and β-catenin mRNA expression. The results showed that mRNA expression of CDK8 and β-catenin was markedly lower in the CDK-siRNA group compared with the other two groups (*P *< 0.01) (Figure 2). However, there was no significant difference in mRNA expression between the scrambled siRNA group and non-siRNA group.

**Figure 3:**
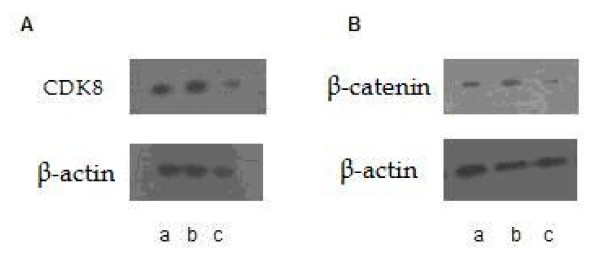
**Representative Western blots of CDK8 and β-catenin expression level in CDK-siRNA transfected HCT116 cells**. 72 h later of CDK8-siRNA transfection of HCT116 cells, protein expression of CDK8 (A) and β-catenin (B) was determined by western blot assay. a: non-siRNA group; b: scrambled siRNA group; c: CDK-siRNA group. Results are given as average value of the gray in three target genes and interal controls from three independent experiments.

Following a 72 h CDK8-siRNA transfection of HCT116 cells, protein expression of CDK8 and β-catenin was determined by western blot assay. As shown in figure 3, CDK8 and β-catenin expression was remarkably reduced in the CDK-siRNA group compared to the other two groups (*P *< 0.01). Similarly, there was no significant difference between the scrambled siRNA group and non-siRNA group.

### Effect of CDK8-siRNA transfection on the growth of HCT116 cells

**Figure 4:**
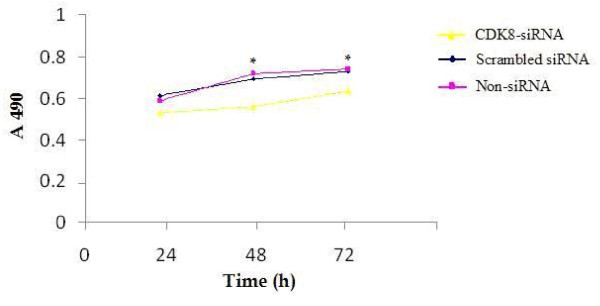
**Cell proliferation activity after transfection of CDK8-siRNA assessed by MTT assay**. Curves of cell growth after transfection for 24, 48 and 72 h by MTT assay. Results are given as means ± SD from three independent experiments. *P *< 0.05.

The cell proliferation of HCT116 cells following 24, 48 and 72 h of transfection was detected by MTT assay. Results showed that the number of viable cells in the CDK8-siRNA group was significantly lower compared to that of the scrambled siRNA and non-siRNA groups 48 and 72 h after CDK8-siRNA transfection (*P *< 0.05) (Figure 4).

### Effect of CDK8-siRNA transfection on the apoptosis and cell cycle of HCT116 cells

**Figure 5:**
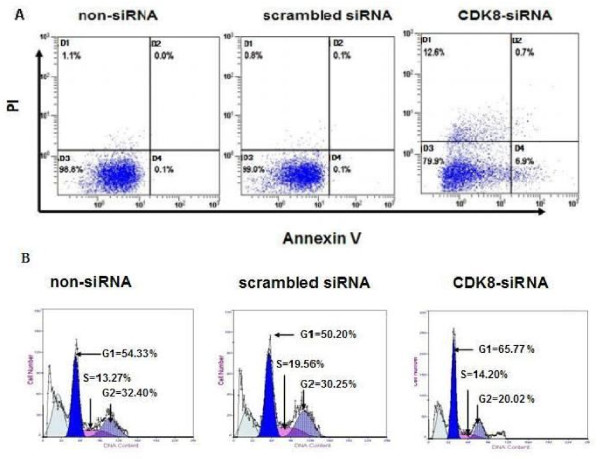
**Effect of CDK8-siRNA transfection on the apoptosis and cell cycle of HCT116 cells**. 48 h after transfection, cell apoptosis (A) and cell cycle (B) were determined by flow cytometry. Quadrants D2-D4 represent necrotic/late apoptotic cells, viable cells, and early apoptotic cells, respectively. Results are given as means ± SD from three independent experiments.

We performed experiments to evaluate the apoptosis and cell cycle of HCT116 cells by CDK8-siRNA. As shown in figure 5, following 48 h transfection, it was indicated that the rate of apoptosis in the CDK8-siRNA group (23.50 ± 1.20%) was significantly higher than that of the scrambled siRNA (4.87 ± 1.48%) and non-siRNA groups (4.77 ± 1.42%) (*P *< 0.01) (Figure 5A). On the other hand, the cell cycle analysis showed that G0/G1 phase of CDK8-siRNA transfected group in a ratio of 65.77 ± 1.17%, was significantly higher than that of scrambled siRNA (50.20 ± 2.43%) and non-siRNA group (54.33 ± 2.55%) (*P *< 0.01) (Figure 5B).

### CDK8 and β-catenin expression in fresh colon tumor and adjacent normal tissues

**Figure 6:**
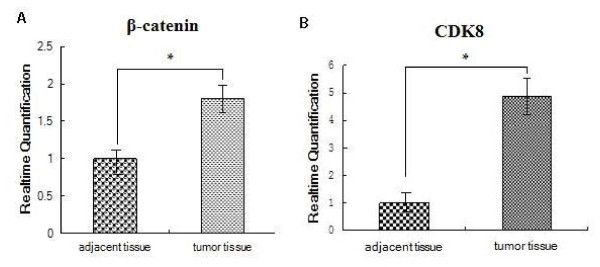
**CDK8 and β-catenin mRNA expression in colon tumor and adjacent normal tissues detected by real-time PCR**. Fresh tumor and corresponding adjacent tissues from 12 patients were resected under sterile conditions and then snapfrozen in liquid nitrogen immediately. 200 mg tissue was taken out from liquid nitrogen and plused 1 ml Trizol when RNA was extaracted. Real-time PCR is performed for the expression levels of CDK8 (A) and β-catenin (B). Results are given as means ± SD from three independent experiments. *P *< 0.05.

To further confirm the expression of CDK8 and β-catenin in colon cancer, we detected the expression of CDK8 and β-catenin in fresh colon cancer tissues and adjacent normal tissues of the same patient. Real-time PCR was adopted to detect the mRNA levels. Results showed that mRNA expression levels of CDK8 and β-catenin in tumor tissues was significantly higher than in adjacent normal tissues (*P *< 0.05) (Figure 6). In addition, the expression of CDK8 was correlated with the expression of β-catenin in both tumor tissues (r = 0.744, *P *< 0.01) and adjacent normal tissues (r = 0.650, *P *< 0.05).

**Figure 7:**
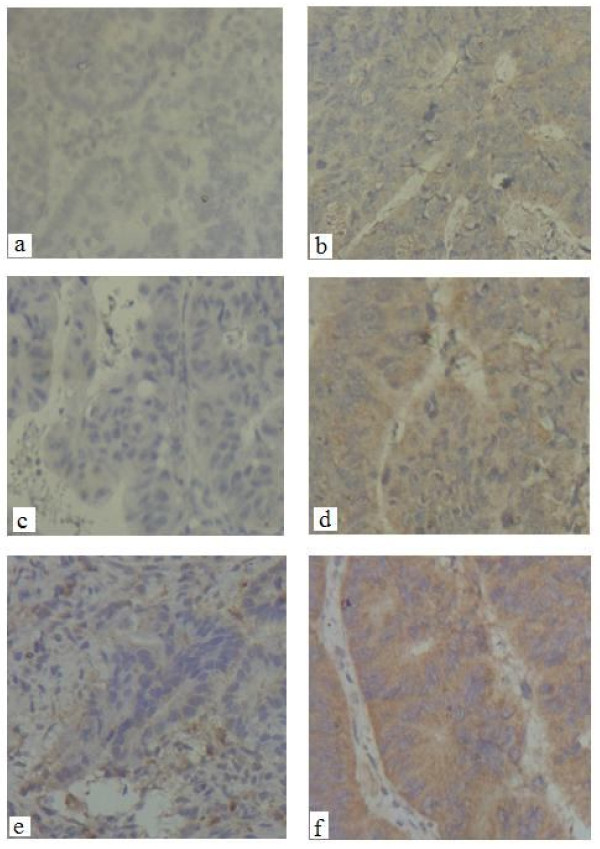
**CDK8 and β-catenin protein expression in colon tumor and adjacent normal tissues detected by IHC**. The expression of CDK8 (left) and β-catenin (right) was stained brown and present in tumor tissue and adjacent normal tissues. Representative sites with negative (a, 400 **X **), moderate positive (c, 400 × ), strongly positive (e, 400 ×) expression of CDK8 and corresponding weakly positive (b, 400 ×), moderate positive (d, 400 ×), strongly positive (f, 400 ×) expression of β-catenin.

Shown by IHC, the positive cells were expressed as brown particles distributed in the cytoplasm of tumor cells (Figure 7). Of the 47 colon tumors, the positive rate of CDK8 and β-catenin was 76.6% (36/47) and 95.7% (45/47). However, in adjacent normal tissues, the positive rate was 21.3% (10/47) and 88.9% (40/47), respectively. The difference between them was statistically significant (*P *< 0.05). As predicted, the expression of CDK8 was also correlated with the expression of β-catenin in both tumor tissues (r = 0.485, *P *< 0.05) and adjacent normal tissues (r = 0.346, *P *< 0.05).

## Discussion

Aberrant activation of the Wnt/β-catenin pathway has been shown to be associated with numerous human cancers [1,2,16]. Previous studies revealed that an abnormality in β-catenin signaling pathway may be responsible for almost all types of colon cancers [4,17]. It has been reported that CDK8 plays a central role in the regulation of β-catenin activation [3,18]. Based on such a background, further exploring of the role of CDK8 and β-catenin in the oncogenesis and progression of colon cancer as well as their correlation, not only provides a broad understanding of the etiology of colon cancer, but also may provide an intervention stategy with CDK8 and β-catenin as a target.

Ron Firestein *et al *[8] found that CDK8 was necessary for the β-catenin-mediated activation of proto-oncogenes. They noted that, in the absence of CDK8, the activity of β-catenin-mediated transcription was significantly decreased, whereas an overexpression of CDK8 could induce proto-oncogene activation [19]. Additionally, Morris and colleagues screened E2F1-dependent apoptotic genes and found that E2F1 could inhibit Wnt/β-catenin activity and CDK8 was the most potential inhibitor of E2F1 [9,19]. Furthermore, CDK8 may also be involved in other signaling pathways. It is reported that CDK8 is a positive co-stimulatory regulator of the expression of p53 gene [20] and p53's downstream gene p21 since the binding of CDK8 to the p53 gene can increase its transcription activity. Furthermore, CDK8 could regulate the Notch signaling pathway [21] and exerted positive regulatory effects on the tumorigenicity related mRNA prolongation [22]. Therefore, CDK8 may be considered to be a proto-oncogene based on the above observations.

To investigate the effects of the activity of β-catenin on colon cancer through CDK8, CDK8 interference was constructed and transfected in colon cancer cells CT116 by the application of siRNA in our study. The alteration of the expression of β-catenin, proliferation, cell apoptosis and cell cycle distribution in HCT116 cells were determined. After silencing CDK8 by siRNA, our results showed that β-catenin expression was markedly lower at the mRNA and protein level, and it is considered that there was a significant association between CDK8 and β-catenin expression. Such results support the claim of Ron Firestein *et al *[8] that only CDK8 play a central role of post-translational modulator of β-catenin in colon cancer. Additionally, it was showed that cell proliferation was reduced after CDK8 blocking using MTT assay. Flow cytometry analysis revealed that the rate of cell apoptosis in the CDK8-siRNA group was markedly higher compared to the control groups, and the majority of cells was in the G0/G1 phase in the CDK8-siRNA group. We suggest that CDK8-siRNA transfection may decrease cell proliferation and facilitate apoptosis of colon cancer cells. Furthermore, the cell cycle arrest after CDK8-siRNA transfection may be related to the reduced transcription activity of β-catenin, since β-catenin can regulate the expression of certain cell cycle-related genes, including survivin and c-myc. However, the exact effect and mechanism on these downstream genes of β-catenin followed with marked reduction of CDK8 needs to be elucidated in future studies. According to our results, it was speculated that the possibility of the regulation of colon cancer through control of CDK8 is theoretically applicable.

To confirm the expression and relationship of CDK8 and β-catenin based on colon cancer tissues, real-time PCR and IHC were performed in our study. As predicted, both CDK8 and β-catenin expression level were markedly higher in tumor compared to adjacent normal tissues. Furthermore, the expression of β-catenin showed positively related to CDK8 expression. Meanwhile, it is reported that the expression of β-catenin was still positive or high in some colon cancer cell lines that have negative expression of CDK8. It is suggested that there might be other factors for regulating the activity of β-catenin such as pancreatic adenocarcinoma up-regulated factor (PAUF) [23] and Delta-like4 (DLL4) [24] expect CDK8. Neverthless, our observations suggested that CDK8-siRNA can effectively inhibit the transcription activity of the β-catenin signaling pathway in colon cancer cells HCT116, thereby resulting in the suppression of cell proliferation and promotion of apoptosis. Further studies would be of interest to determine whether silencing CDK8 and other factors together could amplificate the silencing effect of the β-catenin.

Based on the high specificity of CDK8 to β-catenin, CDK8 may be used as an alternative target in the regulation of colon cancer. Given the number of CDK inhibitors are being applied in clinical practice [25,26], future studies are needed to evaluate the potential power of specific CDK8 inhibitors candidate on the downregulation of β-catenin expression, and subsequently on the inhibition of proto-oncogenes. Our observations demonstrated that the activity of CDK8 is essential to be able to regulate β-catenin-dependent transcription and transformation in colon cancer cells. Accordingly, it is indicated that the intervene stategy targeting CDK8 in colon cancer may be of clinical value.

## Conclusions

In this study, we demonstrated that CDK8 specific siRNA transfection down-regulated the expression of CDK8, which is expressed in a high fraction in colon cancer. We also found out that CDK8 specific siRNA inhibited the proliferation of colon cancer cells, promoted their apoptosis and arrested these cells in the G0/G1 phase. In addition, CDK8 inhibition may be associated with the down-regulation of β-catenin. Our results showed that CDK8 and β-catenin could be promising target in the regulation of colon cancer by the control of β-catenin through CDK8.

## Competing interests

The authors declare that they have no competing interests.

## Authors' contributions

SH, YY, and LW carried out literature research, experimental studies and data acquisition, participated in the study design, and drafted the manuscript. MY and YZ participated in the design of the study and performed the statistical analyses. XZ proposed the study, and participated in its design and coordination and helped to draft, and assisted writing the manuscript. All authors read and approved the final manuscript.
